# Association of National Football League Fan Attendance With County-Level COVID-19 Incidence in the 2020-2021 Season

**DOI:** 10.1001/jamanetworkopen.2022.40132

**Published:** 2022-11-18

**Authors:** Justin Kurland, Wanda E. Leal, Erin M. Sorrell, Nicole Leeper Piquero

**Affiliations:** 1 National Center for Spectator Sports Safety and Security, University of Southern Mississippi, Hattiesburg; 2 Department of Criminal Justice & Criminology, Sam Houston State University, Huntsville, Texas; 3 Center for Global Health Science and Security, Georgetown University, Washington, DC; 4 Department of Microbiology and Immunology, Georgetown University, Washington, DC; 5 Department of Sociology & Criminology, University of Miami, Coral Gables, Florida

## Abstract

**Importance:**

The 2020-2021 National Football League (NFL) season had some games with fans and others without. Thus, the exposed group (ie, games with fans) and the unexposed group (games without fans) could be examined to better understand the association between fan attendance and local incidence of COVID-19.

**Objective:**

To assess whether NFL football games with varying degrees of in-person attendance were associated with increased COVID-19 cases in the counties where the games were held, as well as in contiguous counties, compared with games without in-person attendance for 7-, 14-, and 21-day follow-ups.

**Design, Setting, and Participants:**

This cross-sectional study used data for all 32 NFL teams across the entirety of the 2020-2021 season. Separate daily time-series of COVID-19 total cases and case rates were generated using 7-, 14-, and 21-day simple moving averages for every team and were plotted against the actuals to detect potential spikes (outliers) in incidence levels following games for the county in which games took place, contiguous counties, and a combination. Outliers flagged in the period following games were recorded. Poisson exact tests were evaluated for differences in spike incidence as well across games with different rates of attendance. The data were analyzed between February 2021 and March 2021.

**Exposures:**

Games with fan attendance vs games with no fan attendance, as well as the number of fans in attendance for games with fans.

**Main Outcomes and Measures:**

The main outcome was estimation of COVID-19 cases and rates at the county and contiguous county level at 7-, 14-, and 21-day intervals for in-person attended games and non–fan attended games, which was further investigated by stratifying by the number of persons in fan-attended games.

**Results:**

This included a total of 269 NFL game dates. Of these games, 117 were assigned to an exposed group (fans attended), and the remaining 152 games comprised the unexposed group (unattended). Fan attendance ranged from 748 to 31 700 persons. Fan attendance was associated with episodic spikes in COVID-19 cases and rates in the 14-day window for the in-county (cases: rate ratio [RR], 1.36; 95% CI, 1.00-1.87), contiguous counties (cases: RR, 1.31; 95% CI, 1.00-1.72; rates: RR, 1.41; 95% CI, 1.13-1.76), and pooled counties groups (cases: RR, 1.34; 95% CI, 1.01-1.79; rates: RR, 1.72; 95% CI, 1.29-2.28) as well as for the 21-day window in-county (cases: RR, 1.49; 95% CI, 1.21-1.83; rates: RR, 1.50; 95% CI, 1.26-1.78), in contiguous counties(cases: RR, 1.37; 95% CI, 1.14-1.65; rates: RR, 1.45; 95% CI, 1.24-1.71), and pooled counties groups (cases: RR, 1.41; 95% CI, 1.11-1.79; rates: RR, 1.70; 95% CI, 1.35-2.15). Games with fewer than 5000 fans were not associated with any spikes, but in counties where teams had 20 000 fans in attendance, there were 2.23 times the rate of spikes in COVID-19 (95% CI, 1.53 to ∞).

**Conclusions and Relevance:**

In this cross-sectional study of the presence of fans at NFL home games during the 2020-2021 season, results indicated that fan attendance was associated with increased levels of COVID-19 in the counties in which the venues are nested within, as well as in surrounding counties. The spikes in COVID-19 for crowds of over 20 000 people suggest that large events should be handled with extreme caution during public health event(s) where vaccines, on-site testing, and various countermeasures are not readily available to the public.

## Key Points

**Question:**

Was fan attendance at National Football League (NFL) games during the 2020-2021 season associated with subsequent spikes in COVID-19 cases in-county and in contiguous counties?

**Findings:**

In this cross-sectional study of NFL games attended by a total 1.3 million fans, the presence of large numbers of fans at NFL games was associated with increases in the incidence of COVID-19 cases both in the counties in which these venues were located and contiguous counties. Specifically, NFL games that had 20 000 fans in attendance had 2.23 times the rate of spikes in COVID-19, but NFL games with fewer than 5000 fans in attendance did not generate any spikes.

**Meaning:**

This analysis suggests that in-person attended games during the NFL’s 2020 season were associated with subsequent spikes in COVID-19 cases, and that the spikes were most prominent when attendance was over 20 000 persons.

## Teaser

This cross-sectional study examines the association of in-person attendance at National Football League games during the 2020-2021 season with total cases and case rates of COVID-19 in home and surrounding counties.

## Introduction

When the National Football League (NFL) announced its intentions to play a full season of football in 2020, some thought the decision was devoid of consideration of the health and well-being of the players, coaches, staff members, and even the fans who would attend. However, others wanted live football back as the games would offer a respite from the stress and anxiety that the COVID-19 pandemic had brought to their lives. The NFL, like other professional sport leagues, was dealing with large revenue-based financial losses and a desire to resume play. In an effort to start the season, league officials had to assess how they could safely return 9 months into a worldwide pandemic that had killed hundreds of thousands of Americans.^1,2^

The NFL implemented prevention, mitigation, and surveillance protocols in its facilities, both during team travel and on game days. The design involved continuous team testing, the use of device-recorded interactions between persons, and contact tracing. The totality of implemented measures for players and staff was evaluated, and it appears that the NFL was able to maintain relatively low rates of infection among players and staff.^3^ Because of its care and responsiveness, the NFL was able to carry out the season with almost no serious problems, no game cancellations, and concluded with the Super Bowl in Tampa.

While the extent to which COVID-19 spread throughout the NFL is important, so too is the health safety of the fans that attended games in person. At present, limited research has examined the association between in-person sporting events and COVID-19 cases. For example, Johnson et al^4^ used mathematical models to simulate the periodic influx from outside an immediate community to estimate how in-person sporting events may have factored into the incidence of COVID-19 cases on a college campus. Their models predicted that in-person sporting events could increase COVID-19 cases on a college campus from 25% to 822%. Similarly, Leal et al^5^ found that the number of college football games played was associated with significantly higher COVID-19 cases at the colleges and universities studied. Conversely, Toumi et al^6^ detected no increase in COVID-19 cases after in-person NFL and National Collegiate Athletic Association (NCAA) games. It is important to note that these studies were conducted prior to the widespread implementation of COVID-19 vaccines.

During the 2020-2021 NFL season, 20 out of 32 teams allowed varied numbers of fans to attend some or all home games. These teams had to assess and implement a variety of safety and public health protocols, including approaches to facility sanitation, crowd control, and social distancing. In order to take advantage of the variation in fan attendance across teams, this observational study evaluates the potential concurrence of fan attendance on localized COVID-19 transmission levels.

### Data and Methods

This study followed the Strengthening the Reporting of Observational Studies in Epidemiology (STROBE) reporting guidelines for cross-sectional studies. As all data were obtained from public sources, this study did not constitute human participant research and did not require institutional review board review or exemption as per the US Department of Health and Human Services (45 CFR §46).

### COVID-19 Cases

Daily cumulative US county-level COVID-19 case data (from March 11, 2020, through March 1, 2021) were procured from the *New York Times*’s Coronavirus (COVID-19) Data in the United States GitHub repository.^7^ The data was processed by first taking the reverse cumulative sum to convert it into a daily count series. A novel, conditional interpolation method was developed and applied to handle erroneously deflated daily cases on holidays and inflated daily counts the day following a holiday (commonly referred to as “data dumps”), which occur in some counties but not others, and could lead to false-positive outliers being flagged. A series of additional conditional interpolation algorithms were developed and used to account for counties that failed to report on a given weekend day and then had artificially inflated counts when next reported (references provide additional information on the interpolation method used).^8,9^

### COVID-19 Rates

Residential population data for each of the 219 counties that either had an NFL stadium within them or were contiguous to them were assembled using the 2019 US Census population estimates. Daily rates were then calculated in line with current practice by taking the daily COVID-19 cases for a given county (or group of counties) per 100 000 population.^2^

### NFL Games, Fan Attendance, and Venue Safety Protocols

**Figure 1.**
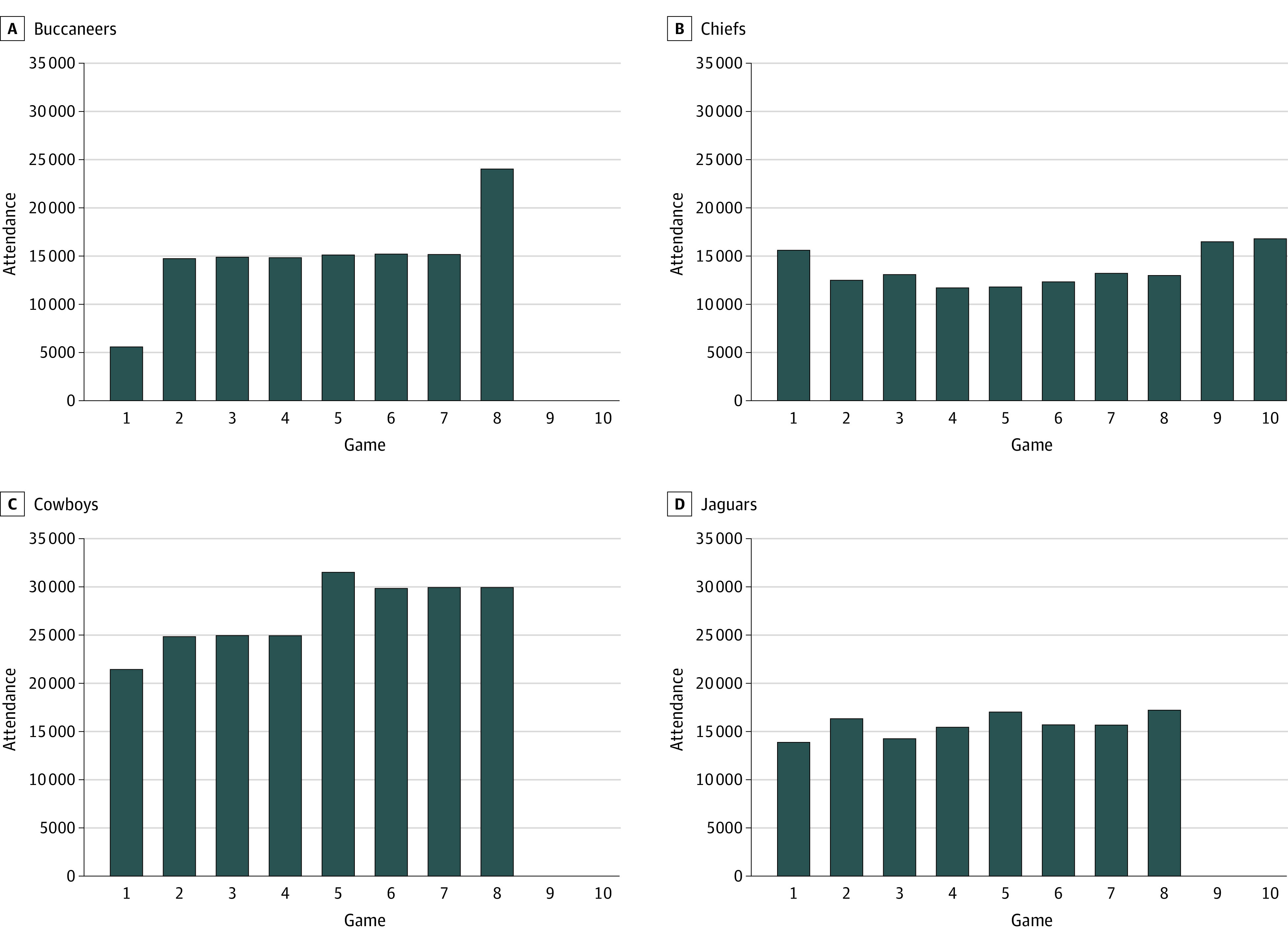
Attendance by National Football League Team That Had Home Games With Fans for the 2020-2021 Season The Chiefs hosted 2 additional home playoff games.

A total of 269 total NFL game dates, locations, and fan attendance were obtained from the Entertainment and Sports Network (ESPN) website. Of these games, 117 were assigned to an exposed group (fans attended) and the remaining 152 games comprised the unexposed group (unattended). Fan attendance ranged from 748 to 31 700 persons (Figure 1) (more details on the dates and attendance numbers for all NFL games can be found in eTable 1 in the Supplement). Team-specific COVID-19 safety protocols may help explain differences in COVID-19 cases but are beyond the scope of this study due to the fact we cannot rule out that stadiums may have implemented safety protocols that were not publicly posted on their website (eTable 2, eAppendix in the Supplement).

### Statistical Analysis

A 3-stage approach to the analysis was adopted. The initial stage, a low-pass filter (LPF) approach, required all permutations of 7-, 14-, and 21-day simple moving averages (SMAs) and their associated 95% CIs to be generated for the counties that host NFL teams, the contiguous counties where many fans likely reside, and a subsequent pooled series for both the host county and those that surround it. This was done for both the raw COVID-19 daily cases and the rates (per 100 000 population). The selection of these particular SMAs was deliberate, as they attempt to mirror the clinically accepted time between exposure to SARS-CoV-2 to symptom onset outlined in the US Centers for Disease Control and Prevention’s Interim Clinical Guidance for Management of Patients with Confirmed Coronavirus Disease, which indicates an incubation period of up to 14 days.^10,11,12,13^

These SMAs and their associated CIs were then plotted against the actual case totals or case rates for each day. The actual COVID-19 daily case totals (or rates) that were above the upper limit of the 95% CI of the 7-, 14-, or 21-day SMAs were flagged as anomalous spikes, as they indicated a significant, positive change from the normal process. This LPF approach is used in signal processing, as a technical trading strategy, in network traffic analysis, and various other areas to detect outliers in univariate time-series data or frequencies.^14,15,16^ A further univariate technique was also implemented, beginning with seasonal and trend decomposition using Loess (STL) to isolate the remainder of a decomposed series (season, trend, and remainder), and then a generalized extreme studentized deviate (GESD) many outlier procedure test to detect anomalies in univariate series.^17,18^ Detection parameters for the latter technique were adjusted to capture only the most egregious anomalies (ie, the Tampa Bay Buccaneers, as they both hosted the Super Bowl and permitted over 20 000 fans to attend [eFigure in the Supplement]). This additional technique was used because there are a multitude of ways to both define (in statistical terms) and evaluate what is considered an outlier or anomaly in the extant time series forecasting and signal processing literature, wherein one technique may suggest that a given point is not an outlier and another may suggest it is. Thus, to provide even stronger empirical evidence in support of the detection of outliers in the period or windows after games, we leveraged 2 entirely different approaches. When both identified a point as an outlier, we could be much more confident that what was flagged as an outlier was indeed an outlier.

**Figure 2.**
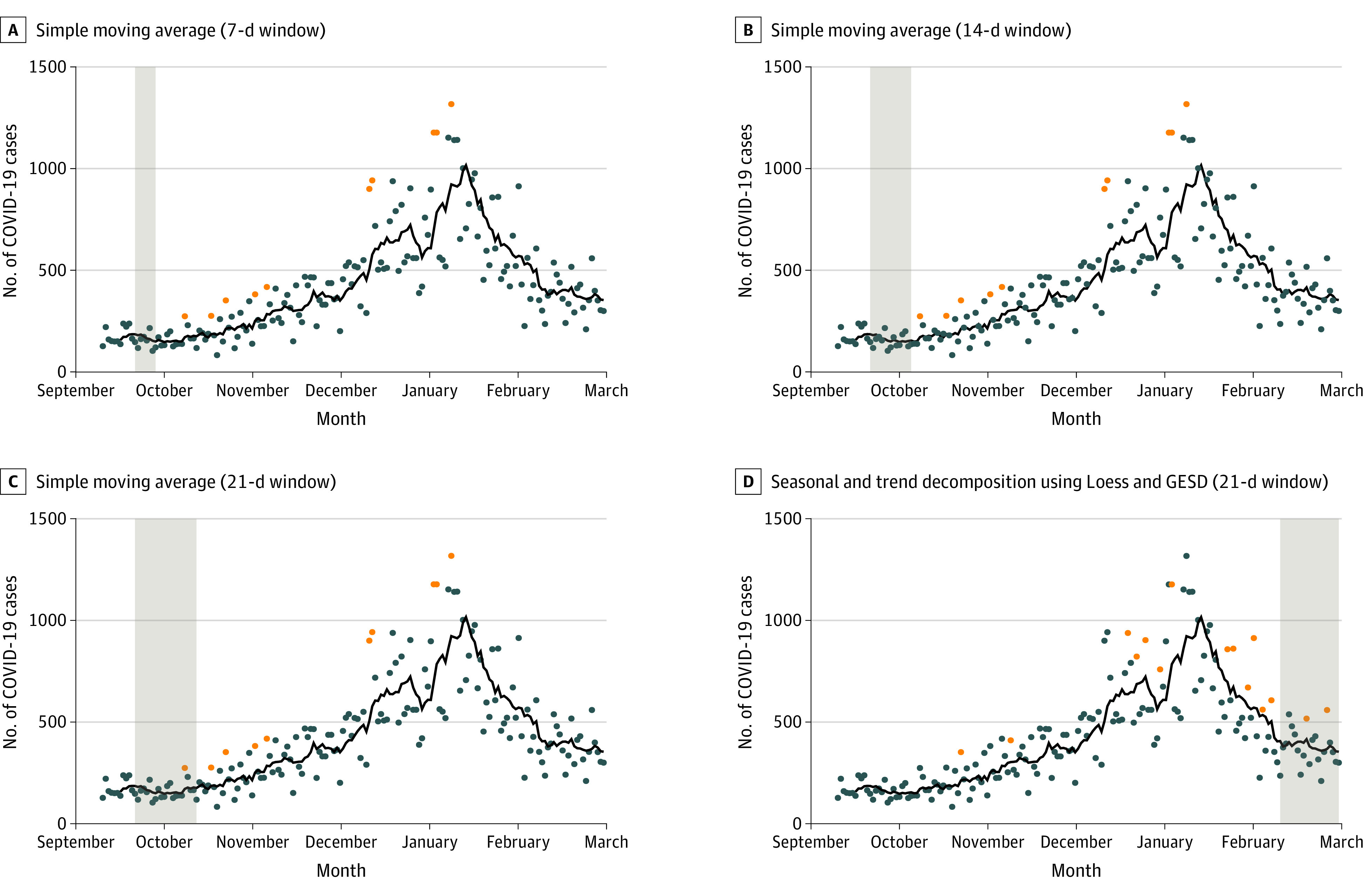
Windows Used for Capturing Outliers for the Period of the Study for the Tampa Bay Buccaneers The black line indicates 7-day simple moving averages; orange points, outliers detected for being above the upper 95% CI; shaded areas, the 7-, 14-, and 21-day periods after all games used to capture flags. Panel D, shows the same window procedure being applied to the outliers that were flagged using the Seasonal and Trend decomposition using Loess with Generalized Extreme Studentized Deviate (GESD) procedure. The Tampa Bay Buccaneers were selected as an illustrative example of the approach because outliers detected within the different windows could be readily visualized for both the earliest game of the season and the final game of the season, the Super Bowl.

The next stage took advantage of the known game dates and locations to set up a series of temporal windows. Three separate windows were used for each respective game to capture flags (outliers) during the expected period from exposure to SARS-CoV-2 to symptom onset and official case recording after games took place. Variance in the onset of symptoms, reporting, and recording underpin the selection of multiple windows. The 7-day window represented the 7 days immediately following a game, a 14-day window the 14 days immediately following a game, and the 21-day window the 21-day period immediately following a game. A total of 807 windows were generated for the total 269 NFL games (351 exposed windows where fan attended games occurred, 456 unexposed windows where no fans were permitted to attend) that took place across the entirety of the 2020-2021 season and used in conjunction with the LPF and the STL outlier detection techniques. The total number of flags within a window were recorded. Hit rates were then calculated by summing all flags captured within a window across all windows for each respective category (Figure 2).

The final step in the analytic process made use of a Poisson exact approach to test the hypothesis that, the presence of fans at NFL football games was associated with a higher occurrence of COVID-19 total cases and case rates in the counties in which the games take place as well as in surrounding counties. We defined λ_1_ as the incidence (outliers) detected per-window total of the exposed group (games with fans); and let λ_2_ be the incidence per-window total of the unexposed group (games with no fans). The research problem was to test, *H_0_: λ_1_ ≤ λ_2_ vs H_1_: λ_1_ ≥ λ_2_*. The test is ideally suited to cases where observations have contrasting durations (eg, 351 vs 456 windows), as tests that rely solely upon the boundary of equality risk inflation of type I error rate because they fail to consider the null space of inferiority.^19^

To test other more specific hypotheses related to spikes in the incidence of COVID-19 cases (and rates), new dose–specific exposed groups were established for those games that had fewer than 5000 fans (15 games), between 5000 and 10 000 fans (32 games), 10 000 and 15 000 fans (46 games), 15 000 and 20 000 fans (15 games), and more than 20 000 fans (9 games). More specifically, because there was variance in game attendance, we took advantage of this to identify a potential threshold tolerance for an acceptable attendance rate for fans that did not appear to be associated with an increase in the incidence of COVID-19 cases and rates. Put differently, the varying number of fans that attended games enabled a more specific set of tests that examined whether a given attendance capacity saw no spikes in the incidence of COVID-19 cases or rates. Data were analyzed with R version 3.6.2 (R Project for Statistical Computing). The threshold for significance was *P* < .05 in 2-sided tests.

## Results

**Figure 3.**
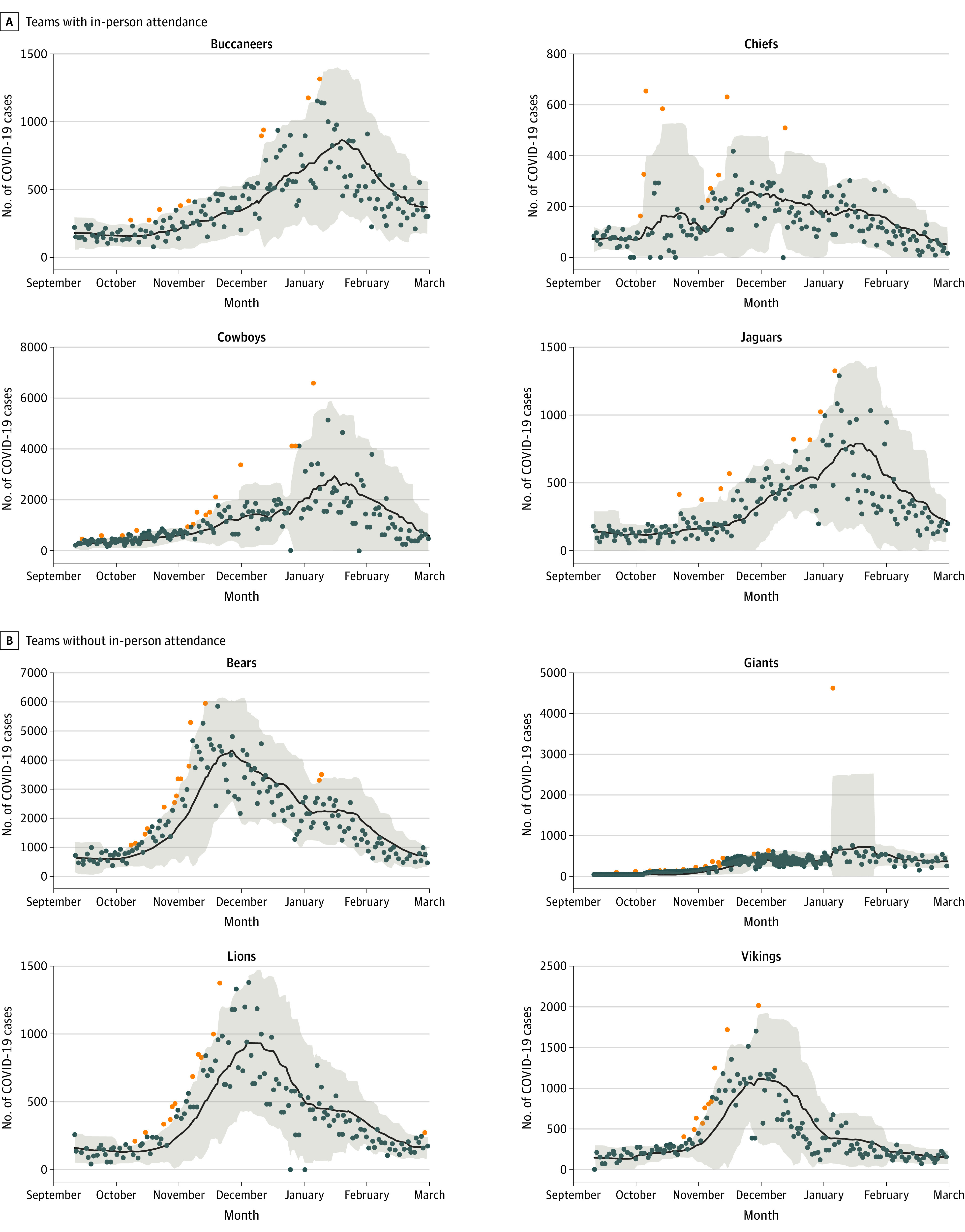
Low-Pass Filter Approach With the 21-Day Simple Moving Average In-County for the Period of the Study for 8 National Football League Teams The black line indicates 7-day simple moving averages; orange points, outliers detected for being above the upper 95% CI; shaded areas, 95% CIs.

Overall, 269 total NFL games (117 with fans and 152 without fans) were included; a total of XX individuals attended. A total of 3456 time-series were generated to capture the overall trend and volatility associated with the incidence of COVID-19 cases and rates both in the county in which NFL stadia are present and those that surround them for the 2020-2021 season, including those that were excluded from the analysis (because of poor recording practices, such as in Houston). These time-series were leveraged to first test whether, on average, a greater number of spikes (outliers) in the incidence of COVID-19 cases and rates occurred in those counties where there were fan-attended games (Figure 3).

The in-county results from the various outlier detection approaches supported that fan attendance at NFL games was associated with episodic spikes in the count of COVID-19 cases and rates with Poisson mean tests for the 21-day SMA, 14-day window in-county (cases: rate ratio [RR], 1.36; 95% CI, 1.00-1.87) and 21-day SMA, 21-day window in-county (cases: RR, 1.49; 95% CI, 1.21-1.83; rates: RR, 1.50; 95% CI, 1.26-1.78) both indicating a significantly greater number of outliers detected.

**Figure 4.**
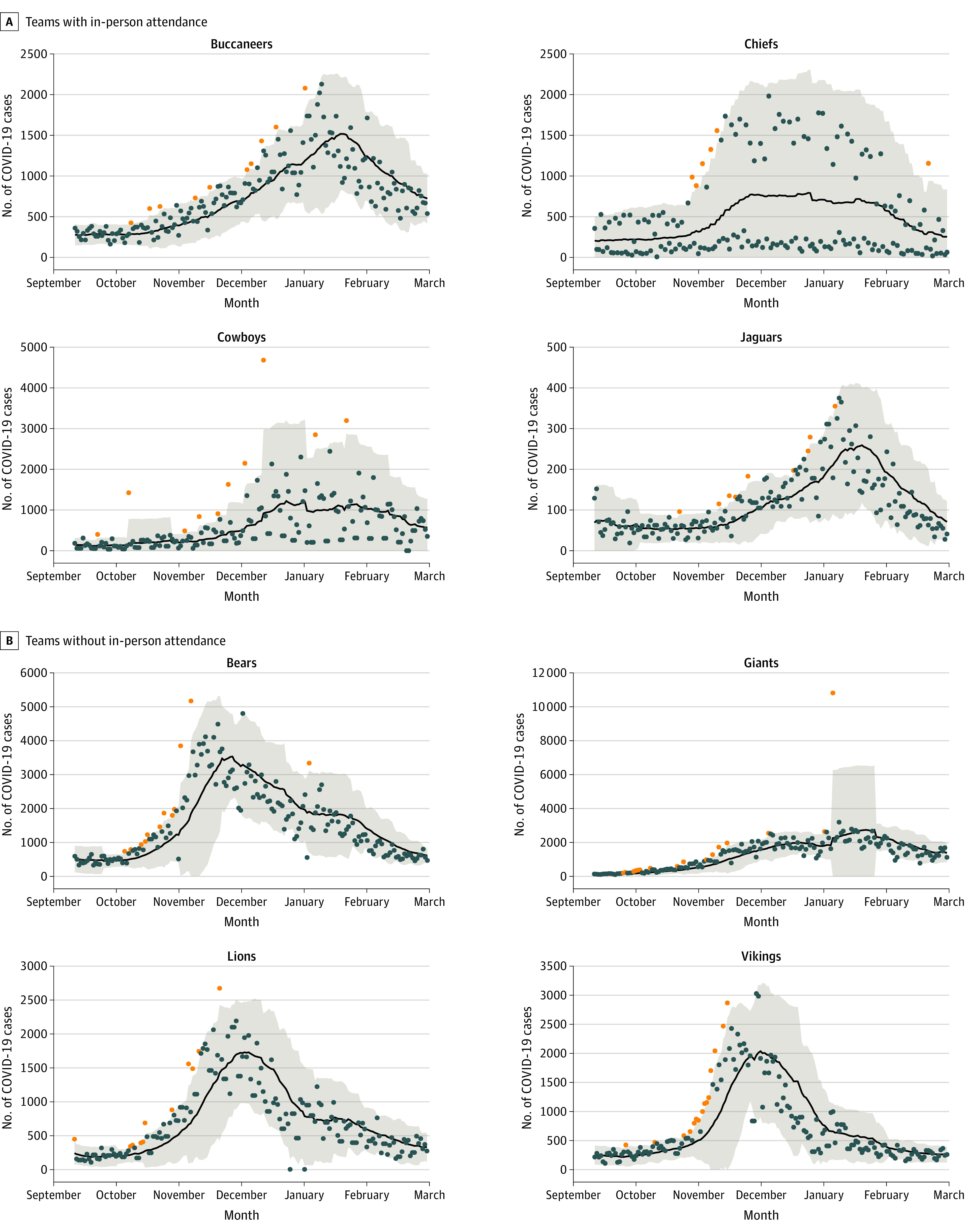
Low-Pass Filter Approach With the 21-Day Simple Moving Averages for Surrounding Counties for the Period of the Study for 8 National Football League Teams The black line indicates 7-day simple moving averages; orange points, outliers detected for being above the upper 95% CI; shaded areas, 95% CIs.

Results for the surrounding counties also provided evidence in support of an increase in the number of spikes (outliers) of COVID-19 cases (and rates) associated with fan-attended games (Figure 4). Poisson mean tests for the 21-day SMA, 14-day window for surrounding counties (cases: RR, 1.31; 95% CI, 1.00-1.72; rates: RR, 1.41; 95% CI, 1.13-1.76) and 21-day SMA, 21-day window for surrounding counties (cases: RR, 1.37; 95% CI, 1.14-1.65; rates: RR, 1.45; 95% CI, 1.24-1.71) both indicating a significantly greater number of outliers detected.

**Figure 5.**
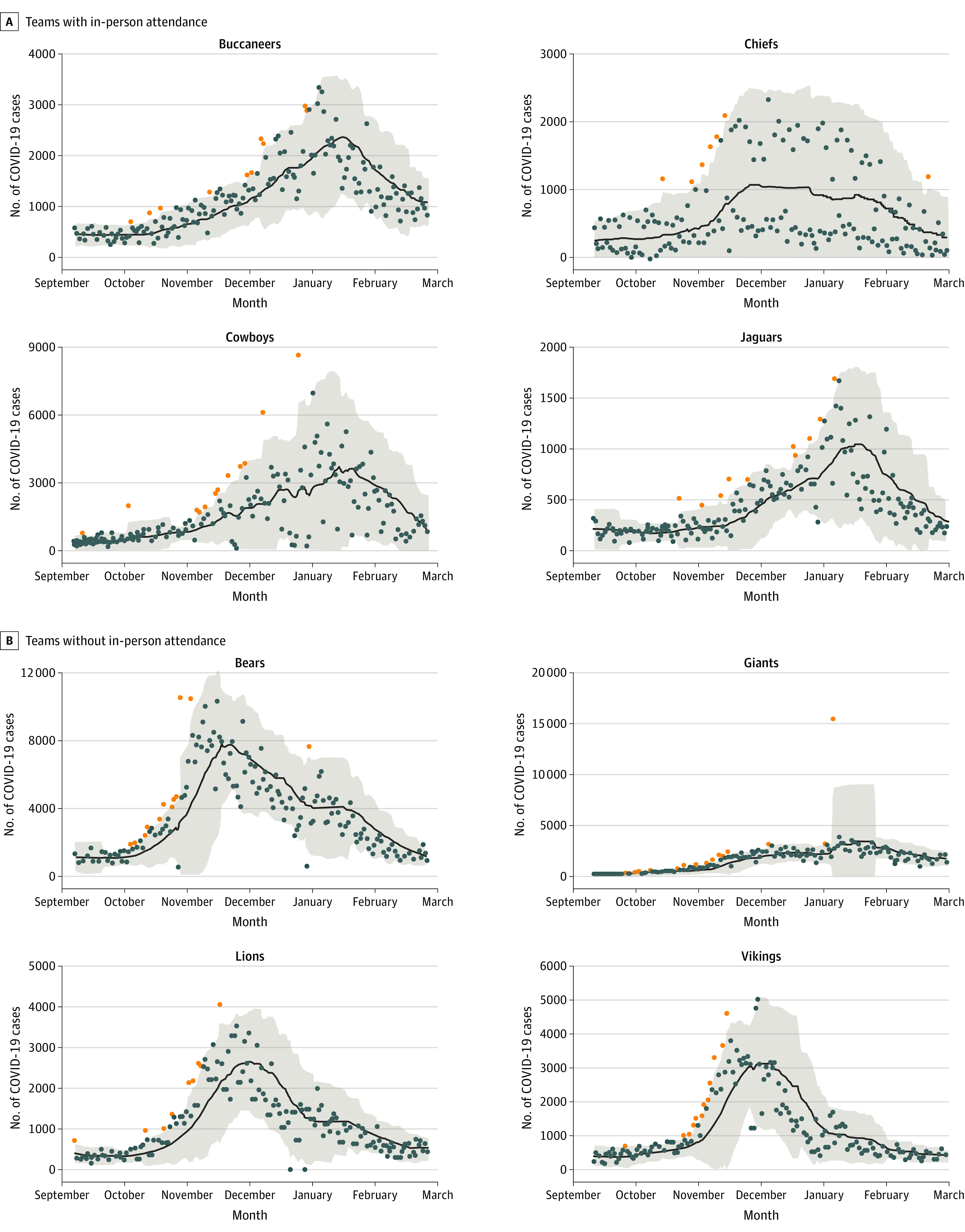
Low-Pass Filter Approach With the 21-Day Simple Moving Averages for In- and Contiguous Counties for the Period of the Study for 8 National Football League Teams The black line indicates 7-day simple moving averages; orange points, outliers detected for being above the upper 95% CI; shaded areas, 95% CIs.

The total county results are reflective of the in-county and surrounding counties analyses, with strong evidence in support of a greater number of spikes (outliers) in the incidence of COVID-19 cases and rates for the fan attended games (Figure 5). Poisson mean tests for the 21-day SMA, 14-day window for COVID-19 case incidence for both in-county and surrounding counties (cases: RR, 1.34; 95% CI, 1.01-1.79; rates: RR, 1.72; 95% CI, 1.29-2.28), and 21-day SMA, 21-day window for surrounding counties (cases: RR, 1.41; 95% CI, 1.11-1.79; rates: RR, 1.70; 95% CI, 1.35-2.15) indicated a significantly greater number of outliers detected.

The total results for the 4 outlier detection techniques and associated hit rates analyses applied to the in-county, surrounding counties, and total pooled counties groupings provide compelling support mirroring the clinically accepted time between exposure to SARS-CoV-2 to symptom onset outlined in the US Centers for Disease Control and Prevention’s Interim Clinical Guidance for Management of Patients with Confirmed Coronavirus Disease, which indicates an incubation period of up to 14 days.^10,11,12,13^ There is a noticeable absence of significant differences in the detection of outliers between the exposed group and unexposed group for the 7-day temporal period immediately following games; however, the majority of tests that used the 14-day and 21-day SMA and LPF technique and the STL and GESD approach (25 out of 36 tests) indicated that the exposed groups were associated with a significant increase in the incidence of spikes in cases and rates of COVID-19 within the counties in which games occurred, the surrounding counties, and the pooled total counties.

We also sought to determine whether a dosage associated with the exposed group provided evidence at the tails of the attendance figures. The results indicated that NFL games that had fewer than 5000 fans in attendance were not associated with spikes in the incidence (either cases or rates) of COVID-19 at any spatial resolution investigated herein. In contrast, the results provided support that games with over 20 000 fans in attendance were associated with spikes in incidence within the county in which the games were played. The 21-day SMA, LPF technique detected a significantly greater incidence of spikes (outliers) in COVID-19 cases and rates in-county (cases: RR, 2.02; 95% CI, 1.26 to ∞; rates: RR, 2.23; 95% CI, 1.53 to ∞). Specifically, the teams that permitted more than 20 000 fans to attend games in person, the Dallas Cowboys and Tampa Bay Buccaneers, experienced double the rate of spikes in the incidence of COVID-19 cases and rates.

## Discussion

In 2020, the NFL resumed competition and in some cases with limited fan attendance, raising the possibility that such events may spread the virus because they would allow potentially infectious individuals to come into close contact with one another for prolonged periods of time. As some teams allowed fans while others did not, this provided an opportunity to investigate the extent to which games attended by fans would be associated with significant changes to COVID-19 cases (or rates) in the subsequent weeks within the context of the 2020 NFL season.

Our results were striking: when fans were allowed to attend games, we found very little evidence of spikes in the first 7-day postgame window, regardless of spatial resolution (eg, in-county, surrounding counties, all counties), which was somewhat expected given the incubation period of the virus variant. However, the results were entirely different for the 14- and 21-day postgame windows. This difference was large and was found for the majority of the comparisons undertaken between cases and rates for in-county, contiguous counties, and pooled results from both groups. Games that allowed fans, regardless of total numbers in attendance, were associated with a much higher rate of COVID-19 spikes compared with games that did not have fans. Further analysis showed that games limiting fans to under 5000 posed less risk while games that permitted more than 20 000 fans posed greater risk.

It is important to note that our study was distinct from and comes to different conclusions than a 2021 study^6^ that examined in-person attendance in NFL and NCAA games and detected no increase in COVID-19 cases in 3 ways. First, Toumi et al^6^ only included 19.1% of NFL games, whereas our study included every game. Second, our study examined both in-county and contiguous county COVID-19 cases whereas Tuomi et al^6^ only considered in-county spread. Third, our study examined the number of fans in attendance whereas Toumi et al^6^ only included a dichotomous measure indicating fan or non–fan attended games. Consideration of these factors may explain the differing results.

This study highlights the importance of a holistic approach to reopening and managing public events during a disease outbreak when vaccines and rapid testing are not readily available. Several lessons from the last 2 years can be applied to how we continue our response to variants of COVID-19 and prepare for the next threat—because there will be another. This study collected data on disease transmission at a time when various control measures were lacking. In the time since our analysis was originally conducted, we have experienced 2 major variants (and subvariants) with increased transmissibility (Delta and Omicron), gained understanding on transmission dynamics, improved testing capacity, implemented nationwide vaccine campaigns for children ages 6 months and older and have improved treatment protocols. These advancements are critical to national and global response and recovery from COVID-19 and should be considered in planning and implementation of future public events. Threats posed by infectious diseases cannot always be predicted but they can be countered by preparation.

### Limitations

Several limitations should be noted. First, it was impossible to assess cause and effect to the fan-attended games and the increase in cases or rates over the ensuing weeks. For example, it is possible that areas with less stringent COVID-19 restrictions, which research has linked to higher incidences of cases and deaths,^20^ were more encouraging of higher fan attendance. As such, these findings should be interpreted as associational and not causal. Second, we cannot determine whether the increases in transmission and cases were due directly to fan attendance at the stadium, to tailgating around the stadium, or to individuals congregating in the homes of family and friends, as it is possible that the city or county’s overall approach to pandemic regulations may have affected the number of cases. Third, because there would be too many variables involved in assessing and linking cases of mortality to fan attendance beyond their simple attendance at a game, we were unable to—and uncomfortable with—using the data or our analysis from a whole-of-league approach. Fourth, some teams had home games where only friends and family attended. These games were not included in the analyses due to data unreliability, as not all teams may have publicly announced the occurrence of these games. Additionally, for those cases where we did not identify a spike in cases, we are unsure what may have contributed to that outcome, such as specific health and safety protocols.

## Conclusions

This cross-sectional study of the presence of fans at NFL home games during the 2020-2021 season indicated that fan attendance was associated with increased levels of COVID-19 in the counties with NFL venues as well as in surrounding counties. The spikes in COVID-19 for crowds of over 20 000 people suggest that large events should be handled with extreme caution during public health events where vaccines, on-site testing, and various countermeasures are not readily available to the public.

## Notes

Supplement. **eTable 1.** NFL Game Dates and Attendance Figures for the 2020-2021 Season **eTable 2.** Stadium Safety Protocols Among Teams That Allowed Fans at Games **eFigure.** Outlier Detection Techniques for the Period of the Study (March 11, 2020–March 1, 2021) for the Tampa Bay Buccaneers (Hillsborough County, Florida). **eAppendix.** Data Sharing Sources
