# Supplementary material for: Association of National Football League Fan Attendance With County-Level COVID-19 Incidence in the 2020-2021 Season
Source: JAMA Netw Open. 2022 Nov 18;5(11):e2240132. doi: 10.1001/jamanetworkopen.2022.40132 (PMC9675007; doi:10.1001/jamanetworkopen.2022.40132)
Supplement: Supplement. — eTable 1. NFL Game Dates and Attendance Figures for the 2020-2021 Season eTable 2. Stadium Safety Protocols Among Teams That Allowed Fans at Games eFigure. Outlier Detection Techniques for the Period of the Study (March 11, 2020–March 1, 2021) for the Tampa Bay Buccaneers (Hillsborough County, Florida). eAppendix. Data Sharing Sources [file jamanetwopen-e2240132-s001.pdf]

Supplemental Online Content

Kurland J, Leal WE, Sorrell EM, Piquero NL. Association of National Football League fan attendance with county-level COVID-19 incidence in the 2020-2021 season. *JAMA Netw Open.* 2022;5(11):e2240132. doi:10.1001/jamanetworkopen.2022.40132

eTable 1. NFL Game Dates and Attendance Figures for the 2020-2021 Season

eTable 2. Stadium Safety Protocols Among Teams That Allowed Fans at Games

eFigure. Outlier Detection Techniques for the Period of the Study (March 11, 2020–March 1, 2021) for the Tampa Bay Buccaneers (Hillsborough County, Florida).

eAppendix. Data Sharing Sources

This supplemental material has been provided by the authors to give readers additional information about their work.

© 2022 Kurland J et al. *JAMA Network Open.*

eTable 1. NFL Game Dates and Attendance Figures for the 2020-2021 Season

Date	Number of Fans in Attendance
Dallas Cowboys	
9/20/2020	21708
10/4/2020	25021
10/11/2020	25147
10/19/2020	25174
11/8/2020	31700
11/26/2020	30048
12/20/2020	30092
12/27/2020	30131
Jacksonville Jaguars	
9/13/2020	14100
9/24/2020	16563
10/18/2020	14513
11/8/2020	15668
11/22/2020	17244
11/29/2020	15926
12/13/2020	15896
12/27/2020	17445
Tampa Bay Buccaneers	
9/20/2020	0
10/4/2020	6383
10/18/2020	15540
11/8/2020	15740
11/23/2020	15730
11/29/2020	15950
12/12/2020	16031
1/3/2021	16009
2/7/2021	24835
Kansas City Chiefs	
9/10/2020	15895
10/5/2020	12729
10/11/2020	13311
11/1/2020	11932
11/8/2020	12073
12/6/2020	12578
12/27/2020	13470
1/3/2021	13240
1/17/2021	16730
1/24/2021	16993
Houston Texans	
9/20/2020	0
10/4/2020	12102
10/11/2020	12413
10/25/2020	12618
11/22/2020	12503
12/6/2020	12316
12/27/2020	12344
1/3/2021	12504
Miami Dolphins	
9/20/2020	11075

© 2022 Kurland J et al. *JAMA Network Open.*

10/4/2020	12369
10/18/2020	10772
11/1/2020	12397
11/15/2020	12751
12/6/2020	12577
12/13/2020	13057
12/20/2020	13354
Tennessee Titans	
9/20/2020	0
10/12/2020	8403
10/18/2020	10166
10/25/2020	10355
11/8/2020	13871
11/12/2020	13949
12/6/2020	13986
12/20/2020	13797
1/10/2021	14029
Indianapolis Colts	
9/20/2020	2500
9/27/2020	7480
10/18/2020	12453
11/8/2020	12200
11/22/2020	12495
11/29/2020	12464
12/20/2020	9992
1/3/2021	9976
Cleveland Browns	
9/17/2020	6000
9/27/2020	6000
10/11/2020	11759
11/1/2020	10972
11/15/2020	10613
11/22/2020	11133
12/14/2020	11974
Cincinnati Bengals	
9/13/2020	0
10/4/2020	6243
10/25/2020	9732
11/1/2020	9712
11/29/2020	10208
12/12/2020	10322
12/21/2020	10249
1/3/2021	10499
Atlanta Falcons	
9/13/2020	0
9/27/2020	0
10/11/2020	0
10/25/2020	7796
11/8/2020	7665
11/29/2020	9124
12/6/2020	8713
12/20/2020	10703
Denver Broncos	

© 2022 Kurland J et al. *JAMA Network Open.*

9/14/2020	0
9/27/2020	5226
10/25/2020	5314
11/1/2020	5231
11/22/2020	5351
11/29/2020	0
12/19/2020	0
1/3/2021	0
Carolina Panthers	
9/13/2020	0
10/4/2020	5120
10/18/2020	5240
10/29/2020	5240
11/15/2020	5815
11/22/2020	5546
12/13/2020	5768
1/3/2021	1500
Pittsburgh Steelers	
9/20/2020	0
9/27/2020	0
10/11/2020	4708
10/18/2020	5260
11/15/2020	5909
12/2/2020	0
12/7/2020	0
12/27/2020	0
1/10/2021	0
New Orleans Saints	
9/13/2020	0
9/27/2020	748
10/12/2020	749
10/25/2020	3000
11/15/2020	5979
11/22/2020	6000
12/20/2020	3000
12/25/2020	3000
1/10/2021	3000
1/17/2021	3750
Arizona Cardinals	
9/20/2020	0
9/27/2020	0
10/25/2020	1200
11/8/2020	4200
11/15/2020	4200
12/6/2020	0
12/20/2020	0
12/26/2020	0
Baltimore Ravens	
9/13/2020	0
9/28/2020	0
10/11/2020	0
11/1/2020	4345
11/22/2020	0

© 2022 Kurland J et al. *JAMA Network Open.*

12/8/2020	0
12/20/2020	0
12/27/2020	0
Washington Football Team	
9/13/2020	0
10/4/2020	0
10/11/2020	0
10/25/2020	0
11/8/2020	3000
11/22/2020	0
12/20/2020	0
12/27/2020	0
1/9/2021	0
Green Bay Packers	
9/20/2020	0
10/5/2020	0
11/1/2020	0
11/15/2020	0
11/29/2020	0
12/6/2020	0
12/19/2020	0
12/27/2020	0
1/16/2021	7439
1/24/2021	7772
Buffalo Bills	
9/13/2020	0
9/27/2020	0
10/19/2020	0
11/1/2020	0
11/8/2020	0
11/29/2020	0
12/13/2020	0
1/3/2021	0
1/9/2021	6772
1/16/2021	6772
Minnesota Vikings	
9/13/2020	0
9/27/2020	0
10/18/2020	0
11/8/2020	0
11/22/2020	0
11/29/2020	0
12/6/2020	0
12/20/2020	0
Los Angeles Chargers	
9/20/2020	0
9/27/2020	0
10/25/2020	0
11/8/2020	0
11/22/2020	0
12/6/2020	0
12/13/2020	0
12/27/2020	0

© 2022 Kurland J et al. *JAMA Network Open.*

San Francisco 49ers	
9/13/2020	0
10/4/2020	0
10/11/2020	0
10/18/2020	0
11/5/2020	0
12/7/2020	0
12/13/2020	0
1/2/2021	0
Chicago Bears	
9/20/2020	0
10/4/2020	0
10/8/2020	0
11/1/2020	0
11/16/2020	0
12/6/2020	0
12/13/2020	0
1/3/2021	0
Los Angeles Rams	
9/13/2020	0
10/4/2020	0
10/26/2020	0
11/15/2020	0
11/29/2020	0
12/10/2020	0
12/20/2020	0
1/3/2021	0
Philadelphia Eagles	
9/20/2020	0
9/27/2020	0
10/18/2020	0
10/22/2020	0
11/1/2020	0
11/30/2020	0
12/13/2020	0
1/3/2021	0
New England Patriots	
9/13/2020	0
9/27/2020	0
10/18/2020	0
10/25/2020	0
11/15/2020	0
11/29/2020	0
12/28/2020	0
1/3/2021	0
Las Vegas Raiders	
9/21/2020	0
10/4/2020	0
10/25/2020	0
11/15/2020	0
11/22/2020	0
12/13/2020	0
12/17/2020	0

© 2022 Kurland J et al. *JAMA Network Open.*

12/26/2020	0
Detroit Lions	
9/13/2020	0
10/4/2020	0
11/1/2020	0
11/15/2020	0
11/26/2020	0
12/13/2020	0
12/26/2020	0
1/3/2021	0
Seattle Seahawks	
9/20/2020	0
9/27/2020	0
10/11/2020	0
11/1/2020	0
11/19/2020	0
12/6/2020	0
12/13/2020	0
12/27/2020	0
1/9/2021	0
New York Giants	
9/14/2020	0
9/27/2020	0
10/18/2020	0
11/2/2020	0
11/15/2020	0
12/13/2020	0
12/20/2020	0
1/3/2021	0
New York Jets	
9/20/2020	0
10/1/2020	0
10/11/2020	0
10/25/2020	0
11/9/2020	0
11/29/2020	0
12/6/2020	0
12/27/2020	0
Notes: The last three games for the San Francisco 49ers were played in the Arizona Cardinals Stadium.	

© 2022 Kurland J et al. *JAMA Network Open.*

eTable 2. Stadium Safety Protocols Among Teams That Allowed Fans at Games

	Prohibited Tailgating	Social Distance for Tailgate	Specified Entry & Exit Points	Specified Entry Time	Mobile Food Order & Pickup Stations	Ready to go Food Options	Directional Lanes	Limiting Retail Capacity
Kansas City Chiefs	No	Yes	Yes	No	Yes	Yes	No	Yes
Dallas Cowboys	No	Yes	No	No	No	No	No	No
Carolina Panthers	Yes	Prohibited tailgating	Yes	No	Yes	No	Yes	No
Denver Broncos	Yes	Prohibited tailgating	Yes	No	No	No	Yes	No
Washington Football Team	Yes	Prohibited tailgating	No	No	No	No	Yes	No
Cleveland Browns	Yes	Prohibited tailgating	Yes	Yes	No	No	Yes	No
Miami Dolphins	Yes	Prohibited tailgating	No	Yes	Yes	No	No	Yes
Pittsburgh Steelers	Yes	Prohibited tailgating	Yes	No	Yes	No	No	No
Green Bay Packers	Yes	Prohibited tailgating	Yes	No	No	Yes	Yes	No
Indianapolis Colts	Yes	Prohibited tailgating	Yes	No	No	No	Yes	No
Baltimore Ravens	Yes	Prohibited tailgating	Yes	No	No	No	No	Yes
Atlanta Falcons	No	Yes	No	No	Yes	No	Yes	Yes
New Orleans Saints	Yes	Prohibited tailgating	Yes	Yes	No	No	No	Yes
Buffalo Bills*	Yes	Prohibited tailgating	Yes	Yes	No	No	No	No
Tennessee Titans	Yes	Prohibited tailgating	No	No	No	No	No	No
Houston Texans	N/A	N/A	N/A	N/A	N/A	N/A	N/A	N/A
Cincinnati Bengals	Yes	Prohibited tailgating	Yes	No	No	No	No	No
Tampa Bay Buccaneers	Yes	Prohibited tailgating	No	No	Yes	No	Yes	No
Arizona Cardinals	No	Yes	Yes	No	No	No	No	Yes
Jacksonville Jaguars	No	Yes	No	No	Yes	No	Yes	No

Notes: Notes: * The Buffalo Bills required a negative COVID-19 test to attend a game; The NFL required that all stadiums require masks, use seating pods, and use mobile tickets. Information in this table was acquired through each team/stadium website. It is possible that stadiums implemented safety protocols and did not post it on their website; Houston Texans did not provide information about the safety protocols they implemented above the required protocols by the NFL; Teams that did not allow fans for any games were not included in this table as

© 2022 Kurland J et al. *JAMA Network Open.*

safety protocols were unnecessary without fans present. The teams that did not allow any fans were: Seattle Seahawks, Detroit Lions, New England Patriots, San Francisco 49ers, Philadelphia Eagles, Los Angeles Rams, New York Giants, New York Jets, Las Vegas Raiders, Chicago Bears, Los Angeles Chargers, and Minnesota Vikings.

© 2022 Kurland J et al. *JAMA Network Open.*

eFigure. Outlier Detection Techniques for the Period of the Study (March 11, 2020–March 1, 2021) for the Tampa Bay Buccaneers (Hillsborough County, Florida).

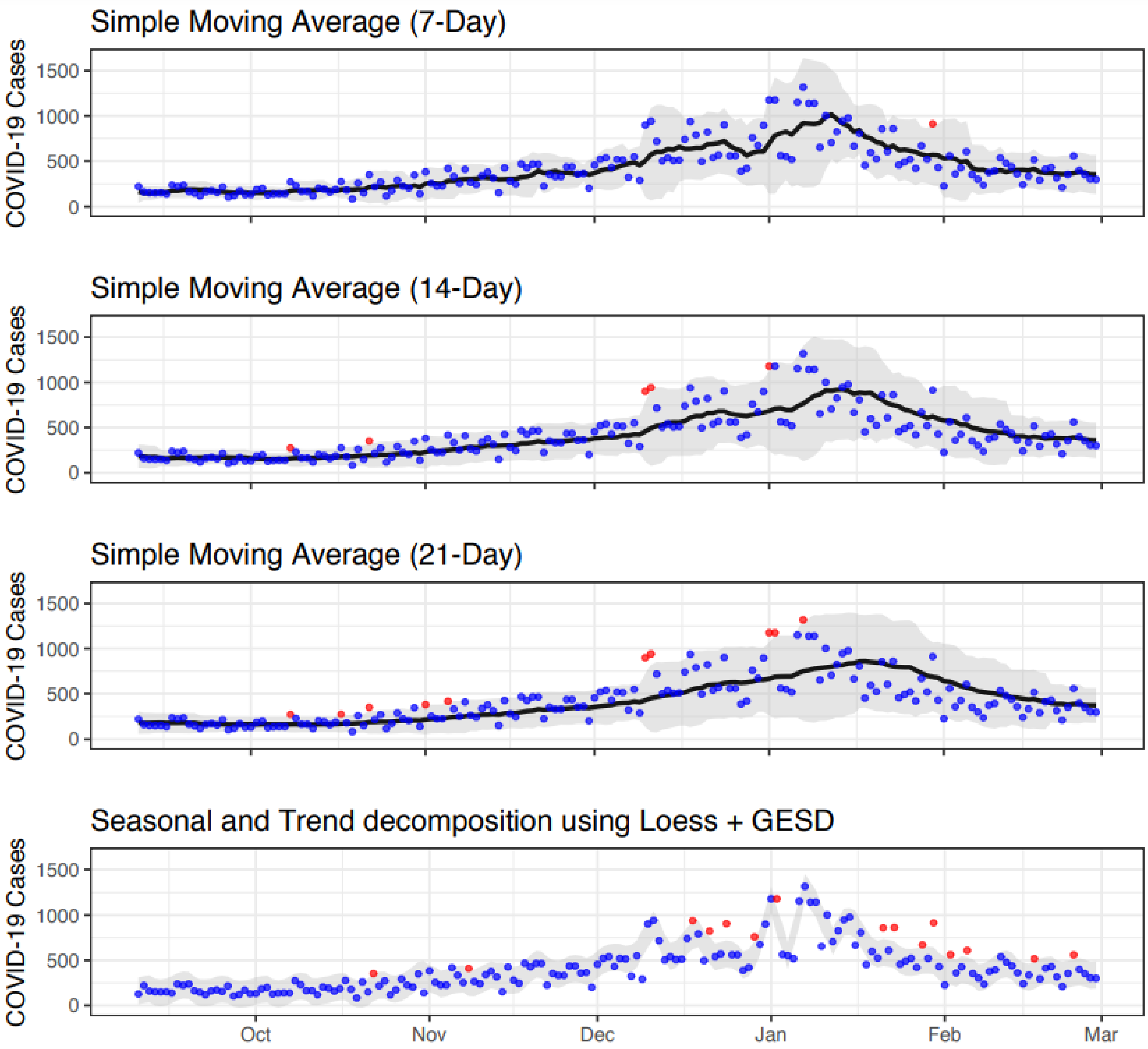

Full caption: The top three panels show the low-pass filter (LPF) approach for each of the 7-, 14-, and 21-day simple moving averages (SMAs). The black line is the SMA, the grey bands are the 95 confidence intervals (CIs). Blue points represent the actual case counts captured within the bands (95 CIs), the red points are the outliers detected above the +95 CI. The bottom panel shows the Seasonal and Trend decomposition using Loess (STL) with Generalized Extreme Studentized Deviate (GESD) procedure. Blue points are the actual cases within the bands (grey), red points are the cases that have been flagged as outliers above the band.

© 2022 Kurland J et al. *JAMA Network Open.*

eAppendix. Data Sharing Sources

Will individual participant data be available (including data dictionaries)?	No individual-level data was used in this study.
What data in particular will be shared?	All daily case and mortality data for the entire period of the study at the county-level for all relevant counties of interest will be shared.
What other documents will be available?	• All original data sources for the study are open access and available to the public.<br>• SARS-CoV-2 cumulative daily case and mortality data are accessible via a repository maintained by the NY Times: https://github.com/nytimes/covid-19-data<br>• Residential population estimates used to generate county-level rates were accessed via a Census API and the 'tidycensus' R package available here: https://github.com/walkerke/tidycensus<br>• NFL game schedule for all games are available here: https://www.nfl.com/schedules/2020/reg1/<br>• NFL attendance figures for games that had fans are available here: http://www.espn.com/nfl/attendance<br>• Stadium-specific SARS-CoV-2 Protocols are available via individual team websites here:<br>• https://www.atlantafalcons.com/stadium/fan-gameday-playbook<br>• https://www.azcardinals.com/stadium/health-and-safety<br>• https://www.baltimoreravens.com/game-day/safe-stadium<br>• https://www.bengals.com/stadium/fan-gameday-playbook<br>• https://www.buccaneers.com/stadium/fan-gameday-playbook<br>• https://www.buffalobills.com/stadium/health-and-safety-guidelines<br>• https://www.chiefs.com/stadium/covid/<br>• https://www.clevelandbrowns.com/stadium/fan-gameday-playbook<br>• https://www.colts.com/game-day/know-before-you-go<br>• https://www.dallascowboys.com/stadium/safe<br>• https://www.denverbroncos.com/stadium/2020/fanexperience/<br>• https://hardrockstadium.com/covid/<br>• https://www.jaguars.com/stadium/<br>• https://www.neworleanssaints.com/fans/fan-gameday-playbook<br>• https://www.nrgpark.com/nrg-park-health-and-safety-reminders/

© 2022 Kurland J et al. *JAMA Network Open.*

	• https://www.packers.com/fans/stay-home-stay-strong<br>• https://www.panthers.com/stadium/2020-fan-policies<br>• https://www.steelers.com/stadium/fan-gameday-playbook<br>• https://www.tennesseetitans.com/stadium/safe-stadium<br>• https://www.washingtonfootball.com/stadium/fedexfieldsafe<br>• Interactive figures for some of the teams are available here:<br>• Indianapolis Colts: https://bookdown.org/justin_b_kurland/indianapolis_colts/<br>• New Orleans Saints:<br>• https://bookdown.org/justin_b_kurland/new-orleans-saints/<br>• Dallas Cowboys:<br>• https://bookdown.org/justin_b_kurland/dallas-cowboys/<br>• Arizona Cardinals:<br>• https://bookdown.org/justin_b_kurland/arizona-cardinals/<br>• Cincinnati Bengals:<br>• https://bookdown.org/justin_b_kurland/cincinnati-bengals/<br>• Cleveland Browns:<br>• https://bookdown.org/justin_b_kurland/cleveland-browns/<br>• Baltimore Ravens:<br>• https://bookdown.org/justin_b_kurland/baltimore-ravens/<br>• Pittsburg Steelers:<br>• https://bookdown.org/justin_b_kurland/pittsburgh-steelers/
When will data be available (start and end dates)?	Immediately following publication; no end date
With whom?	Anyone who wishes to access the data
For what types of analyses?	Any purpose
By what mechanism will data be made available?	Data are available indefinitely at (https://data.mendeley.com/datasets/rzmw4p4kbh/draft?a=01dffca3-69aa-412d-a7cf-73865f83e91c)

© 2022 Kurland J et al. *JAMA Network Open.*
